# Whole genome analysis of clouded leopard species reveals an ancient divergence and distinct demographic histories

**DOI:** 10.1016/j.isci.2022.105647

**Published:** 2022-12-09

**Authors:** Madeline G. Bursell, Rebecca B. Dikow, Henrique V. Figueiró, Olga Dudchenko, Joseph P. Flanagan, Erez Lieberman Aiden, Benoit Goossens, Senthilvel K.S.S. Nathan, Warren E. Johnson, Klaus-Peter Koepfli, Paul B. Frandsen

**Affiliations:** 1 Department of Plant and Wildlife Sciences, Brigham Young University, Provo, UT 84602, USA; 2 Data Science Lab, Office of the Chief Information Officer, Smithsonian Institution, Washington, DC 20560, USA; 3 Center for Species Survival, Smithsonian Conservation Biology Institute, National Zoological Park, Front Royal, VA 22630, USA; 4 The Center for Genome Architecture, Department of Molecular and Human Genetics, Baylor College of Medicine, Houston, TX, USA; 5 Center for Theoretical Biological Physics, Rice University, Houston, TX, USA; 6 Houston Zoo, Inc., 1513 Cambridge, Houston, TX 77030, USA; 7 UWA School of Agriculture and Environment, The University of Western Australia, Crawley, WA 6009, Australia; 8 Departments of Computer Science and Computational and Applied Mathematics, Rice University,Houston, TX, USA; 9 Broad Institute of MIT and Harvard, Cambridge, MA, USA; 10 Shanghai Institute for Advanced Immunochemical Studies, Shanghai Tech University, Shanghai, China; 11 Sabah Wildlife Department, Kota Kinabalu, Sabah, Malaysia; 12 Organisms and Environment Division, Cardiff School of Biosciences, Cardiff, UK; 13 Danau Girang Field Centre, c/o Sabah Wildlife Department, Kota Kinabalu, Sabah, Malaysia; 14 The Walter Reed Biosystematics Unit, Museum Support Center MRC-534, Smithsonian Institution, Suitland, MD, USA; 15 Walter Reed Army Institute of Research, Silver Spring, MD, USA; 16 Loyola University Maryland, Baltimore, MD, USA; 17 Smithsonian-Mason School of Conservation, George Mason University, Front Royal, VA 22630, USA

**Keywords:** Canine genetics, Genomics

## Abstract

Similar to other apex predator species, populations of mainland (*Neofelis nebulosa*) and Sunda (*Neofelis diardi*) clouded leopards are declining. Understanding their patterns of genetic variation can provide critical insights on past genetic erosion and a baseline for understanding their long-term conservation needs. As a step toward this goal, we present draft genome assemblies for the two clouded leopard species to quantify their phylogenetic divergence, genome-wide diversity, and historical population trends. We estimate that the two species diverged 5.1 Mya, much earlier than previous estimates of 1.41 Mya and 2.86 Mya, suggesting they separated when Sundaland was becoming increasingly isolated from mainland Southeast Asia. The Sunda clouded leopard displays a distinct and reduced effective population size trajectory, consistent with a lower genome-wide heterozygosity and SNP density, relative to the mainland clouded leopard. Our results provide new insights into the evolutionary history and genetic health of this unique lineage of felids.

## Highlights

• New whole genome assemblies are generated for the two species of clouded leopard
• Genome-wide diversity analyses confirm lower genetic diversity in the island species
• Ancient divergence between clouded leopard species predates that of leopard and lion
• Demographic analyses reveal distinct historical trajectories of Ne for each species

## Graphical abstract

**Figure:**
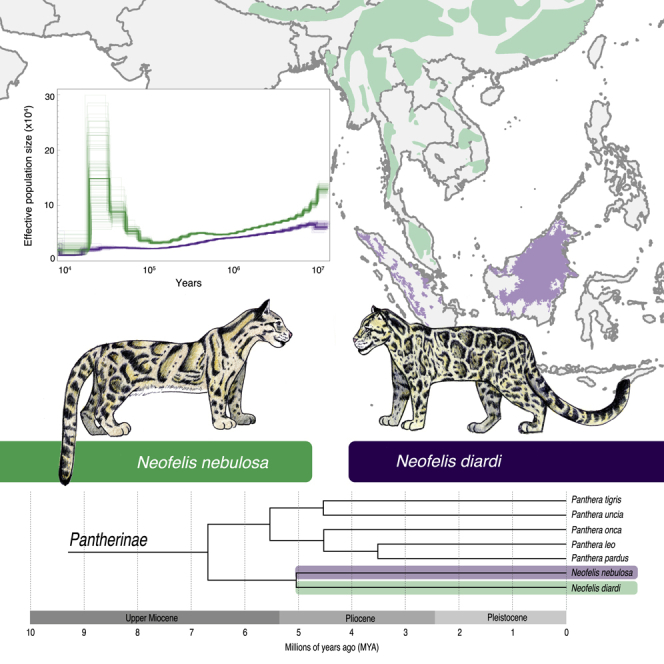

## Introduction

In 1821, naturalist Edward Griffith provided the first description to western science of a clouded leopard based on a skin specimen he classified as *Felis nebulosa*.^1^ Two years later, Georges Cuvier, from a skin provided by Pierre-Médard Diard from Sumatra, described a second species of clouded leopard, *Felis macroscelis*.^2^ After examination of skulls from several different felid species in 1867, John Edward Gray assigned clouded leopards to the genus *Neofelis*,^3^ and these were eventually subsumed into a single species*, Neofelis nebulosa*, a taxonomic designation that was followed for more than a century.^4^^,^^5^ However, in 2006, clouded leopards were once again divided into two distinct species based on evidence from mitochondrial and nuclear DNA sequences, microsatellite and cytogenetic variation, and comparisons of pelage and skull features that all evidenced two deeply divergent and reciprocally monophyletic lineages.^6^^,^^7^^,^^8^^,^^9^ These lineages were formally recognized as the mainland clouded leopard, *N. nebulosa*, distributed in mainland South and Southeast Asia, and the Sunda (or Diard’s) clouded leopard, *Neofelis diardi*, found in Borneo and Sumatra.^6^^,^^7^^,^^8^^,^^10^ More recently, a comprehensive evaluation of felid taxonomy affirmed this reclassification of two species and suggested that further subspecific distinctions among island populations of Sunda clouded leopards should be considered in the future.^11^

Taxonomists have used biochemical and molecular genetic studies to indicate that *Neofelis* is sister to the *Panthera* lineage of cats, which includes the jaguar, lion, leopard, snow leopard, and tiger, and that these two lineages comprise the subfamily Pantherinae, the earliest diverging clade among the extant Felidae.^12^^,^^13^ Previous molecular dating analyses suggest that *Neofelis* and *Panthera* diverged ∼5 million years ago (Mya) during the Late Miocene/Early Pliocene.^12^^,^^13^^,^^14^ Because of their highly elusive natures, the ecology and habitat requirements of clouded leopards are not well known. However, clouded leopards appear to predominantly inhabit primary and secondary evergreen tropical rainforests but have also been found in other habitats such as mangrove swamps.^15^^,^^16^ Like other felids, clouded leopards are hypercarnivorous, hunting a variety of terrestrial and arboreal vertebrate prey and possessing a dentition fitting such a diet, including the longest canines relative to body size among all extant felids. With long tails, broad feet and short legs that position their bodies with a low center of gravity, clouded leopards are excellent climbers, and are thought to use trees primarily as resting sites.^17^ Based on analyses of communication behavior, clouded leopards appear to be solitary for most of the year except during a short mating season, similar to other solitary felid species.^17^^,^^18^

Like many other terrestrial apex predators around the world, populations of the two clouded leopard species are declining and becoming fragmented throughout their respective ranges. Mainland and Sunda clouded leopards are listed as vulnerable on the IUCN Red List of Threatened Species, with mature individuals in the wild estimated to be 3,700–5,580 and 4,500, respectively.^19^^,^^20^ The major threats to both species include habitat loss caused by deforestation for agricultural use, overhunting for pelts, and poaching associated with the illegal wildlife trade.^21^^,^^22^ As a result of these threats, mainland clouded leopards have become locally extinct throughout their former range. As for the global *ex situ* population, there are 375 mainland clouded leopards housed in 108 institutions in 33 countries, especially in Europe and North America, and only ∼56% of the pedigree of this population is known.^23^

The decline and fragmentation of clouded leopard populations raises concerns about the erosion of genetic diversity, increased probability of inbreeding, and the loss of adaptive potential. Empirical studies of genetic diversity using modern molecular genetic or genomic techniques are urgently needed for both wild and *ex situ* populations, but to our knowledge, no such studies have yet been completed, beyond the modeling of landscape connectivity to generate predictions of genetic diversity in Sunda clouded leopards in Sabah state, Borneo.^24^ Reference genomes generated from species of conservation concern provide a first step toward assessing genome-wide genetic diversity, inbreeding, and historical demography, and facilitate the analysis of further genomic data collected from population samples.^25^ Here, we present the first *de novo* genome assemblies for mainland and Sunda clouded leopards and evaluate their genome-wide divergence, diversity, and historical trends in effective population size.

## Results and discussion

### Whole genome sequencing, assembly, and annotation

We generated genome assemblies from one captive mainland clouded leopard (*N. nebulosa*) and one wild Sunda clouded leopard (*N. diardi*) by sequencing paired-end and mate-pair libraries to a cumulative depth of 36.7x and 34.7x, respectively (see STAR Methods). We assembled sequences *de novo* for both species with the MaSuRCA genome assembler.^26^ For the mainland clouded leopard, we also performed Hi-C proximity ligation sequencing and assembly to generate chromosome-length scaffolds.^27^ The contig and scaffold N50s for the mainland clouded leopard assembly are 76.4 kbp and 147 Mbp, respectively, and 48.9 kbp and 1.39 Mbp, respectively, for the Sunda clouded leopard assembly (see Tables S1 and S2). The contiguity of these assemblies is comparable to those of other felids including the lion, *Panthera leo,* (scaffold N50: 136 Mbp)^28^ and the Iberian lynx, *Lynx pardinus* (scaffold N50: 1.52 Mbp).^29^ Across 4,104 conserved mammalian genes,^30^ the assemblies of the mainland and Sunda clouded leopard had Benchmarking Universal Single-Copy Orthologs (BUSCO)^31^ completeness scores of 95.6 and 95.8%, respectively, indicating high gene completeness. Repetitive elements constituted 33.27% of the mainland clouded leopard genome and 32.91% of the Sunda clouded leopard genome. Homology-based gene prediction using gene annotations from human,^32^ domestic dog (ROS_Cfam_1.0, NCBI *Canis lupus familiaris* Annotation Release 106), and domestic cat as references revealed 23,398 protein-coding genes for *N. nebulosa* and 23,193 for *N. diardi*.^33^ These estimates are similar to the number of genes found in the high quality *Felis_catus*_9.0 assembly, which contains 24,546 genes.^33^

### Evolutionary history

We investigated the divergence and evolutionary history of clouded leopards within the context of felid evolution by estimating a time-scaled phylogeny using the newly generated clouded leopard genomes and those from 11 other felid species along with the spotted hyena, which was used for rooting the topologies (see STAR Methods for accession numbers and citations). We aligned 4,104 single-copy orthologs^30^ (9.63 Mb) from each species and estimated phylogenies. Initial trees were estimated using maximum likelihood in IQ-TREE v.1.6.12^34^ with a concatenated supermatrix (see Figure S1A) and a multispecies coalescent species tree was estimated using ASTRAL-III^35^ with individual maximum likelihood gene trees as input (see Figure S1B). Both approaches yielded identical topologies within Pantherinae, with mainland and Sunda clouded leopards forming a sister clade to all *Panthera* species, consistent with previous studies that used smaller (∼22.8–150 kb) multilocus datasets.^12^^,^^13^ Phylogenetic analysis of nearly complete mitochondrial genomes, including the first such genome reported for the Sunda clouded leopard, yielded a similar deep split between *Neofelis* and *Panthera* (see Figure S2). However, relationships among species in the latter genus were discordant relative to the nuclear species tree, particularly regarding the placement of the snow leopard. Such discordance in mitochondrial phylogenies has previously been ascribed to sex-biased asymmetries in dispersal, hybrid sterility, and gene flow via historical hybridization in felids.^13^ Although previous studies have noted signatures consistent with numt contamination in published mitochondrial felid genomes via segments of unusual divergence,^13^ we found no such evidence of numt contamination in our newly assembled clouded leopard mitochondrial genomes using the methods outlined in Li et al. 2016.^13^

Next, we used the supermatrix alignment, the species tree topology inferred with ASTRAL-III, and 11 fossil-based calibration priors and secondary priors for node ages to estimate the time to most recent common ancestor (TMRCA) among the 13 felid species in our dataset using a relaxed molecular clock implemented in MCMCTree from the PAML 4.8 package.^36^ We found that mainland and Sunda clouded leopards diverged around 5.1 Mya (95% credibility interval or CI = 3.8–6.5 Mya) (Figure 2A). Although there is a wide interval of uncertainty around the point estimate, this age places the divergence of these species between the Messinian stage of the Late Miocene and the Zanclean stage of the Early Pliocene, coinciding with a highly dynamic period of sea level changes, including one of the largest marine transgressions (+50 m) at the Miocene-Pliocene boundary, ∼5.3 Mya.^37^^,^^38^ Periods of marine transgressions would have facilitated the isolation of Sundaland from mainland Asia, assuming a model in which an ancestral population of clouded leopards was already distributed in the two regions. Our mean divergence age of 5.1 Mya is much earlier than previous estimates of 1.41 Mya and 2.86 Mya derived using smaller mitochondrial and nuclear datasets,^6^^,^^14^ but is consistent with phylogenetic and historical biogeographic analyses based on combined fossil, morphological, and molecular evidence showing that a majority of pantherine lineages diversified during the Miocene and that the earliest splits among the lineages, including *Neofelis*, occurred in Asia.^39^

**Figure 1:**
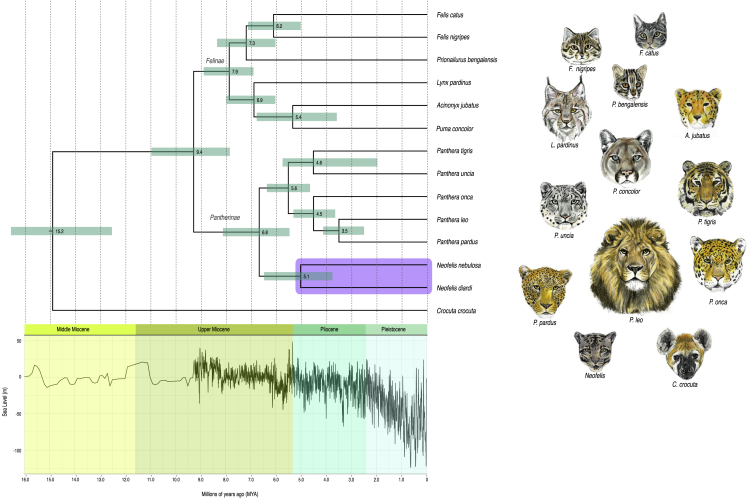
Phylogenetic relationships and divergence times of clouded leopards relative to other felids Time-scaled nuclear phylogeny of 13 felid species, which was rooted using the spotted hyena (*Crocuta crocuta*) as the outgroup (see STAR Methods). Tree topology inferred using ASTRAL-III and divergence times were estimated using MCMCTree, along with eleven secondary priors (see Table S3). Teal horizontal bars represent 95% confidence intervals. Purple bar represents the genus *Neofelis*. The lower figure shows changes in sea level (m) over the last 16 million years.^37^ See also Figures S1 and S2.

**Figure 2:**
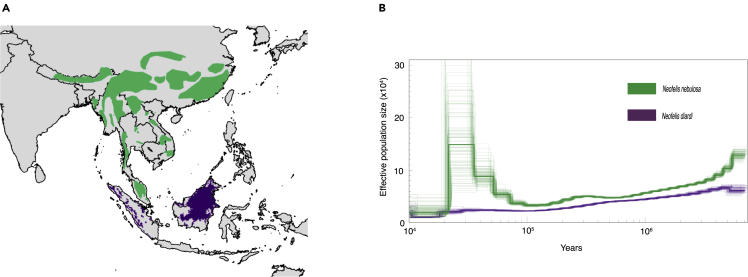
Geographic distribution and inferred demographic history of mainland and Sunda clouded leopards (A) Geographical distribution of mainland (green) and Sunda (violet) clouded leopards based on data from the IUCN.^19^^,^^40^ (B) Trajectories of effective population size (*Ne*) of mainland (green) and Sunda (violet) clouded leopards estimated using the PSMC model. *Ne* trajectories were assessed with 100 bootstrap replicates (lighter lines around darker line) and scaled using a generation length of 7.0 years for *N. nebulosa* and 7.3 years for *N. diardi* and a mutation rate of 2.22 × 10^-9.^^41^^,^^42^ See also Figure S3.

The estimated divergence time between the mainland and Sunda clouded leopards is consistent with the intraspecific or interspecific ages of other forest-dependent mammal or vertebrate taxa that diversified between mainland Asia and Sundaland.^38^^,^^43^ The divergence between the two clouded leopard species predates the intraspecific divergences observed in other felid species with mainland Asia and Sundaic lineages,^44^ as well as the sister pair of the leopard (*Panthera pardus*) and lion (*P. leo*) at 3.5 Mya (95% CI = 2.5–4.2 Mya; Figure 1), which show more ecological and morphological differences. Despite being morphologically similar, our results suggest the split between mainland and Sunda clouded leopards is among the oldest intrageneric divergences among the extant Felidae. The split between *Neofelis* and *Panthera* is estimated at ∼6.8 Mya (95% CI = 5.5–8.2 Mya), which falls within the range of ages (5.5–8.3 Mya) estimated for the same node in a recent phylogenomic analysis of the extinct scimitar-toothed cat (*Homotherium latidens*) in relation to other extant felid species.^45^

### Demographic history

Given the deep divergence age we found between the two species of clouded leopards and because the mainland clouded leopard occupies a wider geographic range on mainland Asia relative to the insular Sunda clouded leopard, we predicted that the historical trajectory of effective population size (*Ne*) for the two species would be distinct and show an overall lower mean *Ne* for the latter species, as species might be expected to have smaller effective population sizes and lower genetic variation compared to mainland lineages.^46^^,^^47^ As predicted, the Sunda clouded leopard exhibited an overall reduced *Ne* throughout its history relative to the mainland clouded leopard when estimated using the pairwise sequentially Markovian coalescent model (PSMC)^48^ (Figure 2A). We note that the genome of the Sunda clouded leopard presented here is derived from a sample collected from a wild individual in the Malaysian state of Sabah in Borneo. Although the total estimated census size for this species is estimated to be ∼4,500 across both Borneo and Sumatra based on niche modeling analyses, these same analyses suggest a census size across suitable (primarily protected) habitats in Borneo of ∼3,800 individuals, but only ∼700–1,000 individuals in Sabah state, depending on the information input source used for the modeling.^24^^,^^49^ Moreover, Sunda clouded leopards are thought to be patchily distributed,^24^^,^^50^which could have reduced their effective population size in more recent times.

We also tested multiple alternative mutation rates and generation lengths for comparison (see Figure S3). Our PSMC results suggest an effective population size of 3,400–18,859 for the Sunda clouded leopard and 20,048–148,718 for the mainland clouded leopard during the last 100,000 years. To compare effective population sizes for each species throughout their history, we estimated the mean effective population sizes of 13,335 and 57,255 for the Sunda and mainland clouded leopard respectively (Figure 2A). We observed a notable increase in *Ne* beginning around 80 Kya in the mainland clouded leopard, which is usually interpreted as a signal of population expansion.^51^ However, this could also be caused by admixture among formerly isolated and structured populations.^52^^,^^53^ As our mainland clouded leopard genome is derived from a zoo animal with a pedigree that includes an unknown founder history, we cannot exclude the possibility of an admixture event in this individual’s history. Nonetheless, the separate *Ne* trajectories suggest long-term isolation between these two species.

### Genomic diversity

**Figure 3:**
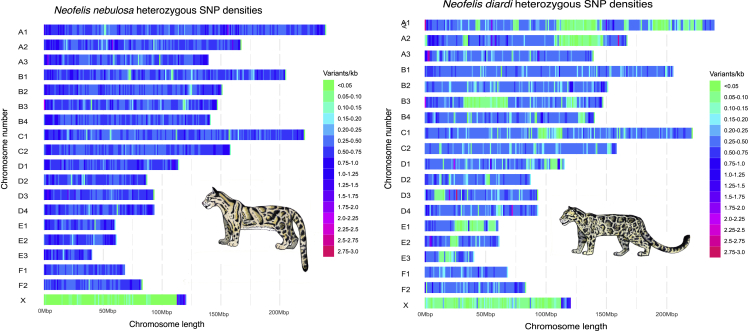
Genetic diversity in mainland and Sunda clouded leopards Density of SNVs across chromosome-length scaffolds of *N. nebulosa*(left) and *N. diardi* (right), based on the chromosome-length assembly of the former species.

We evaluated and visualized the density of single nucleotide variants (SNVs) across the genomes of mainland and Sunda clouded leopards to understand how genetic diversity is distributed between the two species^54^ (Figure 3A). To better compare the location of SNVs in both genomes, we produced chromosome-level assemblies from the short-read assemblies of both species (see STAR Methods). We observe large blocks of low density in the Sunda clouded leopard on chromosomes A1, A2, and B3, but also several shorter regions of high density on, e.g., chromosomes A3, D3, D4, and E2. The SNV density plotted across the mainland clouded leopard was less variable (Figure 3). We estimated lower autosome-wide heterozygosity in *N. diardi* (0.00049) than in *N. nebulosa* (0.00052). Compared to other felid species for which autosome-wide heterozygosity has been estimated, clouded leopards have estimates of genetic variation comparable to that observed in cheetah (*Acinonyx jubatus*) and lower than most other species within the subfamily of Pantherinae, with the exception of the snow leopard.^45^ The lower heterozygosity in the Sunda clouded leopard is consistent with the lower effective population size estimated from the PSMC analyses.

The mainland clouded leopard sample was taken from a North American *ex situ* population, which included a founder size of 18 individuals.^55^ However, gene diversity estimates of descendants based on the pedigree suggest a founder genome equivalent (FGE) as low as four individuals (FGE = 4.14).^55^ Establishing compatible pairs of mainland clouded leopards of breeding age in captivity is extremely challenging, which has in some cases resulted in line breeding within the *ex situ* population. Consequently, the North American Species Survival Plan for mainland clouded leopards has been “facing a demographic crisis” because of the challenges of pairing breeding-age males and females.^56^ Despite best efforts in maintaining genetic diversity in captive-bred populations, some inbreeding is still likely to occur and should be considered in this analysis. Of interest, a large chromosomal inversion was identified in the genome of another captive individual of *N. nebulosa*, a feature that warrants further investigation in the context of the captive breeding program (https://www.dnazoo.org/post/cloudy-with-a-chance-of-leopards).

As populations of clouded leopards continue to dwindle, understanding their individual genetic health and evolutionary and demographic histories has become crucial to their long-term sustainability. Here, we observe that mainland and Sunda clouded leopards have differing demographic histories that reveal a separation of populations and an average lower effective population size for the latter species. We also estimate a new mean divergence date of 5.1 Mya between *N. nebulosa* and *N. diardi*, which corresponds to episodes of marine transgressions that may have promoted the vicariance between populations on mainland Asia and Sundaland, as has been shown in other mammal species. The Sunda clouded leopard genome exhibits more regions of lower SNV density, which correlates with a smaller population size. In addition, this species has less average heterozygosity than observed in the mainland clouded leopard genome. These findings provide a valuable foundation for conservation managers of both captive and wild clouded leopards because they establish key differences in genetic variation between the two species.^56^ Future studies should look to discover additional unique genetic differences between mainland and Sunda clouded leopards that were acquired after a 5.1 Mya divergence. It would also be beneficial to examine a larger number of individuals from both species, thereby granting a more complete picture of genetic health and demographic history.

### Limitations of the study

This study presents the first chromosome-length genome assembly for the mainland clouded leopard. However, unlike the Sunda clouded leopard, which was wild caught, the mainland clouded leopard sample was derived from a captive bred individual, which could affect analyses of genetic diversity. Despite this, we hope that this work will positively contribute to ongoing captive breeding programs for clouded leopards and form a foundation for future studies with multiple samples from wild individuals.

## STAR★Methods

### Key resources table

**Table:** 

REAGENT or RESOURCE	SOURCE	IDENTIFIER
**Biological samples**		

*Neofelis nebulosa* whole blood sample	This study	Smithsonian National Zoological Park-Conservation Biology Institute (NZP-CBI)
*Neofelis diardi* whole blood sample	This study	Sabah Wildlife Department, Kota Kinabalu, Sabah, Malaysia

**Critical commercial assays**		

QIAGEN DNeasy Blood&Tissue Kit	Psomagen	N/A

**Deposited data**		

Figshare deposited data for main text and supplemental analyses	This study	https://doi.org/10.25573/data.c.5990545
*Neofelis nebulosa*sequence reads	This study	GenBank: SRR13774417
*Neofelis nebulosa* sequence reads	This study	GenBank: SRR13774416
*Neofelis nebulosa* assembly	This study	GenBank: PRJNA555324
*Neofelis nebulosa* assembly	This study	https://www.dnazoo.org/assemblies/Neofelis_nebulosa
*Neofelis nebulosa* sequence reads	This study	GenBank: SRX7041771
*Neofelis nebulosa* sequence reads	This study	GenBank: SRX7041772
*Neofelis nebulosa* sequence reads	This study	GenBank: SRX7041774
*Neofelis diardi* sequence reads	This study	GenBank: SRR13774415
*Neofelis diardi* sequence reads	This study	GenBank: SRR13774414
*Neofelis diardi* assembly	This study	GenBank: PRJNA555324
*Felis catus* assembly	Montague et al.^33^	GenBank: PRJNA16726
*Panthera tigris* assembly	N/A	https://www.dnazoo.org/assemblies/Panthera_tigris
*Panthera onca* assembly	Made by DNA zoo	https://www.dnazoo.org/assemblies/Panthera_onca
*Panthera uncia* assembly	Made by DNA zoo	https://www.dnazoo.org/assemblies/Panthera_uncia
*Panthera pardus* assembly	N/A	https://www.dnazoo.org/assemblies/Panthera_pardus
*Panthera leo* assembly	Armstrong et al.^28^	GenBank: PRJNA556895
*Acinonyx jubatus* assembly	N/A	https://www.dnazoo.org/assemblies/Acinonyx_jubatus
*Crocuta crocuta* assembly	Made by DNA zoo	https://www.dnazoo.org/assemblies/Crocuta_crocuta
*Lynx pardinus* assembly	Abascal et al.^29^	GenBank: PRJEB12609
*Felis nigripes*assembly	N/A	GenBank: PRJNA399394
*Prionailurus bengalensis* assembly	https://pubmed.ncbi.nlm.nih.gov/33305796/	GenBank: PRJDB7724
*Puma concolor* assembly	N/A	https://www.dnazoo.org/assemblies/Puma_concolor
*Homo sapiens* assembly	Human Genome Project	GenBank: PRJNA31257
*Canis lupus* assembly	N/A	GenBank: PRJNA615959

**Software and algorithms**		

MaSuRCA v3.2.8	Zimin et al.^26^	http://www.genome.umd.edu/masurca.html
assembly_stats v0.1.4	Trizna,^57^	https://github.com/MikeTrizna/assembly_stats
BUSCO v3.0.2	Simão et al.^31^	https://busco.ezlab.org
RepeatMasker v4.0.9	Smit et al.^58^	https://www.repeatmasker.org/RepeatMasker/
Bowtie 2 v2.3.5	Langmead and Salzberg,^59^	http://bowtie-bio.sourceforge.net/bowtie2/index.shtml
SAMtools v1.9	Li et al.^60^	https://sourceforge.net/projects/samtools/files/samtools/
BCFtools v1.9	Li,^61^	https://samtools.github.io/bcftools/
PSMC v0.6.5	Li and Durbin,^48^	https://github.com/lh3/psmc
FASconCAT	Kück and Meusemann,^62^	https://www.zfmk.de/en/research/research-centres-and-groups/fasconcat
ASTRAL-III v5.7.3	Zhang et al.^35^	https://github.com/smirarab/ASTRAL
GeMoMa v1.7.1	Keilwagen et al.^63^	http://www.jstacs.de/index.php/GeMoMa
GATK v3.8.1.0	McKenna et al.^64^	https://gatk.broadinstitute.org/hc/en-us
Picard v2.20.6	Picard,^65^	https://broadinstitute.github.io/picard/
TrimGalore v0.6.4	Krueger,^66^	https://github.com/FelixKrueger/TrimGalore
VCFtools v0.1.16	Danecek et al.^67^	http://vcftools.sourceforge.net
Bioawk v1.0	Bioawk,^68^	https://github.com/lh3/bioawk
IQ-TREE v1.6.12	Nguyen et al.^34^	http://www.iqtree.org
MAFFT v7.407	Katoh et al.^69^	https://mafft.cbrc.jp/alignment/software/
PAML v4.9	Yang,^36^	http://abacus.gene.ucl.ac.uk/software/paml.html
FigTree v1.4.4	FigTree,^70^	http://tree.bio.ed.ac.uk/software/figtree/
Ragout v2.3	Kolmogorov et al.^71^	https://github.com/fenderglass/Ragout
Cactus v2019.03.01	Armstrong et al.^72^	https://github.com/ComparativeGenomicsToolkit/cactus
snpden_plot	Figueiró,^73^	https://github.com/henriquevf/snpden_plot

### Resource availability

#### Lead contact

Further information can be requested via the lead contact, Paul B. Frandsen (paul_frandsen@byu.edu).

#### Materials availability

This study did not generate any new reagents.

### Experimental model and subject details

#### Sample acquisition

A whole blood sample was collected into a 3.0 mL BD Vacutainer EDTA tube (Becton Dickinson, USA) from a 15-year old male mainland clouded leopard (“Sa Ming”, studbook # 1434, NZP-CBI accession # 114394, DOB: 03/29/2009) during a routine veterinary check-up in 2014 as permitted under the Smithsonian’s National Zoo and Conservation Biology Institute Captive Bred Wildlife registration. The sample was stored at −80°C until genomic DNA extraction. This individual is part of the Smithsonian’s National Zoological Park-Conservation Biology Institute (NZP-CBI) clouded leopard conservation breeding program. A whole blood sample from a 3-year old wild male Sunda clouded leopard was collected into a 3.0 mL Vacutainer EDTA tube after the animal was surrendered to the Sabah Wildlife Department, Kota Kinabalu, Sabah, Malaysia underAccess License no JKM/MBS.1000-2/2 (391) and Export License no JKM/MBS.1000-2/3 JLD.2 granted by the Sabah Biodiversity Council. The sample was stored at −80°C at the Sabah Wildlife Health, Genetic and Forensic Laboratory in Kota Kinabalu until shipment to the Smithsonian National Zoological Park-Conservation Biology Institute.

### Method details

#### DNA extraction and whole-genome sequencing

We extracted genomic DNA from the two whole blood samples of the two clouded leopard species using the Qiagen DNeasy Blood & Tissue Extraction Kit (Qiagen, Valencia, California), following the manufacturer’s protocols. Genomic DNA quality and quantity were evaluated with a double-stranded DNA High-Sensitivity Assay Kit (Invitrogen, Waltham, Massachusetts) and Qubit 2.0 DNA fluorometer (Life Technologies, Carlsbad, California). The genomic DNAs were then delivered to Psomagen, Inc. (Rockville, Maryland) for genomic library preparation and sequencing. Genomic DNA quantity and integrity were re-assessed using Picogreen fluorometry (Life Technologies, Carlsbad, California) and the Genomic DNA ScreenTape assay (Agilent Technologies, Santa Clara, California). Two 350-bp insert size libraries were prepared for each sample using the Illumina TruSeq DNA PCR-free kit (Illumina, San Diego, California). In addition, one 3 kb-insert size mate-pair library for each sample was prepared using the Nextera Mate Pair Library Preparation Kit (Illumina, San Diego, California). All libraries were quality checked using an Agilent Tapestation 4150 instrument and then sequenced on an Illumina HiSeq 2500 sequencer with 101 bp paired-end reads.

### Quantification and statistical analysis

#### Whole-genome assembly and annotation

We trimmed the adapters from the raw read data using TrimGalore.^66^ Further trimming or cleaning prior to assembling Illumina reads is not encouraged when using the MaSuRCA assembler.^26^ We assembled sequencing reads from both the mainland and Sunda clouded leopard *de novo* with the MaSuRCA^26^ assembler using the default parameters for Illumina-only assemblies. We further scaffolded the *N. nebulosa* assembly using Hi-C data generated by DNA Zoo to produce a chromosome-length genome assembly. A whole blood sample for *in situ* Hi-C preparation was donated by a female mainland clouded leopard named Rhu during a routine veterinary check-up in 2015 and provided to us by Houston Zoo. The sample was used to extract peripheral blood mononuclear cell (PBMCs) that were crosslinked and stored at −80°C. The PBCM pellet was used to prepare an *in situ* Hi-C library.^74^ The resulting libraries (NCBI SRX7041771, SRX7041772, SRX7041774) were sequenced using the Illumina NextSeq 500 and HiSeq X Ten instruments. The Hi-C data was aligned to the draft reference assembly for *N. nebulosa* using Juicer^75^ and assembled to chromosome-length using 3D-DNA^27^ and Juicebox Assembly Tools.^76^ The resulting genome assembly is available at https://www.dnazoo.org/assemblies/Neofelis_nebulosa. We assembled the mitochondrial genomes using a single paired-end library from each species using MitoFinder v1.4.1 with the--metaspades option.^77^^,^^78^

We aligned the *Neofelis diardi* short-read assembly to the Hi-C assembly using Cactus v2019.03.01^72^ and used the resulting hal alignment file as input for scaffolding with Ragout v2.3.^71^ This resulted in chromosome assignments for the short-read genome and we used the Ragout-scaffolded assembly for variant calling and PSMC (see details below).

We used assembly_stats v.0.1.4^57^ to generate scaffold and contig statistics for our assemblies. We then cleaned our assemblies by filtering out scaffolds below 500 bp and renaming the remaining scaffolds sequentially using Bioawk v1.0.^68^ To assess the completeness of our assemblies we used Benchmark Universal Single-Copy Orthologs (BUSCO v3.0.2) to search our genomes for 4,104 mammalian orthologous genes included in the OrthoDB v.9 database.^30^^,^^31^ We masked repetitive regions using Repeatmasker and the Carnivora repeat database.^58^ Both genomes were annotated using GeMoMa v1.7.1,^63^which implements a homology-based gene prediction algorithm, with the human (GRCh38.p13, NCBI *Homo sapiens* Updated Annotation Release 109.20210514),^32^ domestic dog (ROS_Cfam_1.0, NCBI *Canis lupus familiaris* Annotation Release 106), and domestic cat (Felis_catus_9.0, NCBI *Felis catus* Annotation Release 104)^33^ used as references.

#### Read alignment and variant calling

We used TrimGalore to remove reads with a PHRED score < 20.^66^ We then used Bowtie2 v2.3.5^59^ “very-sensitive-N 1-I 100-X 600-phred33” to align reads from *N. nebulosa* and *N. diardi* to the Hi-C assembly and scaffold assembly of these species, respectively. BAM files were indexed and sorted using Samtools v1.9,^60^ and duplicate reads were marked using Picard v2.20.6.^65^ For each genome, we also estimated average coverage from the resulting bam files using Samtools v1.9. We used GATK v.3.8.1.0 RealignerTargetCreator and GATK IndelRealigner to improve the alignments by realigning reads in regions that have indels.^64^ Finally, we ran GATK HaplotypeCaller with the “—emitRefConfidence GVCF –variant_index_type LINEAR –variant_index_parameter 128000” commands to produce variant call format (.vcf) files.^79^

#### Inference of historical effective population size with PSMC

We used the *N. nebulosa* Hi-C assembly and the Ragout chromosome-level assembly for *N. diardi* to analyze the demographic histories of both clouded leopards with the Pairwise Sequentially Markovian Coalescence (PSMC) model.^48^ We used Samtools mpileup to generate a bcf file using the fastas and cleaned BAM files from the GATK analysis as input. We used Bcftools call^61^ and the mindepth (1/3 of the average coverage) and maxdepth (2X the average coverage) to call variants and generate a fastq file for both species. We then used PSMC to convert the fastq files to psmcfa format and execute PSMC with the parameters “-N25-t15-r5-p "4+25∗2+4+6.” We plotted with the psmc_plot.pl script with a mutation rate of 2.22e-9^42^ and a generation length of 7.0 years for *N. nebulosa* and 7.3 years for *N. diardi*.^41^ We also performed bootstrapping of both trajectories with 100 additional rounds of PSMC. Additional mutation rates and generation lengths were tested and plotted (Figure S3).

#### Dated phylogeny reconstruction

We downloaded available assemblies for *Felis catus* (NCBI PRJNA16726), *Felis nigripes* (NCBI PRJNA399394), *Prionailurus bengalensis* (NCBI PRJDB7724), *Lynx pardinus* (NCBI PRJEB12609), *Acinonyx jubatus* (DNA Zoo https://www.dnazoo.org/assemblies/Acinonyx_jubatus), *Puma concolor* (DNA Zoo https://www.dnazoo.org/assemblies/Puma_concolor), *Panthera tigris* (DNA Zoo https://www.dnazoo.org/assemblies/Panthera_tigris), *Panthera uncia* (DNA Zoo https://www.dnazoo.org/assemblies/Panthera_uncia), *Panthera onca* (DNA Zoo https://www.dnazoo.org/assemblies/Panthera_onca), *Panthera leo* (NCBI PRJNA556895), *Panthera pardus* (DNA Zoo https://www.dnazoo.org/assemblies/Panthera_pardus), and *Crocuta crocuta* (DNA Zoo https://www.dnazoo.org/assemblies/Crocuta_crocuta). Along with the HiC assembly for *Neofelis nebulosa* and the Ragout-scaffolded assembly for *Neofelis diardi*, we ran BUSCO v3.0.2 on the 12 downloaded assemblies with the--long flag against the 4,104 mammalian orthologous genes included in the OrthoDB v.9 database.^30^^,^^31^ We concatenated the BUSCO results for each species into individual gene files and aligned them using MAFFT v7.407.^69^ After this step, we used a supermatrix and a multispecies coalescence approach to estimate phylogenies. For the supermatrix approach, the aligned gene files were concatenated into a supermatrix with the FASconCAT.pl script.^62^ We then used IQ-TREE v2.0.8^34^ to estimate an optimal partitioning scheme and nucleotide substitution models using the relaxed clustering algorithm followed by model selection with ModelFinder (option-m mfp). Finally, we used IQ-TREE v 2.0.8^34^ to estimate the maximum likelihood tree with 1,000 ultrafast bootstraps (-bb 1000) and 25 individual tree searches. We chose the tree with the highest maximum likelihood which was visualized with FigTree v1.4.4.^70^ For the multispecies coalescence approach, we estimated individual maximum likelihood trees from each of the individual 4,028 aligned genes with IQ-TREE v2.0.8.^34^ We then used these gene trees as input to ASTRAL-III^35^ to estimate a multi-species coalescent species tree, including local posterior probability support values.

#### MCMCTree

We estimated average genomic divergence times among species using the concatenated supermatrix alignment. Node ages were calculated using the supermatrix and the tree topology obtained from the ASTRAL-III^35^ analyses with the MCMCTree program within PAML v4.9.^36^ The relaxed molecular clock model was implemented with the independent lognormal model of rate evolution^80^ with a root age ranging between 7.91Mya and 16.3Mya. We have chosen HKY85 as the evolutionary model (alpha = 0.02, ncatG = 4). BDparas = 1 1 0.1, kappa gama = 6 2, alpha_gamma = 1 1, rgene_gamma = 1 6.89, sigma2_gamma = 1 10. After a burn-in period of 10,000 generations, the MCMC algorithm was sampled every 10th generation until 20,000 samples of divergence time parameters were obtained, totaling 210,000 generations. We used 11 secondary priors to calibrate node ages (Table S3).

#### Single nucleotide variant (SNV) density plots

Plots showing the density of heterozygous SNVs were generated for each clouded leopard species based on the final .vcf file using VCFtools v0.1.16^67^ “snpden” function with a window size of 1Mb using a custom script in R (https://github.com/henriquevf/snpden_plot).^73^ SNV densities were then plotted onto the scaffolded assemblies of each species and then scaled to SNVs per Kbp.^54^

## Data Availability

• Genomic data have been deposited to Genbank and are publicly available as of the date of publication. Accession numbers are listed in the key resources table. Supplemental data, VCFs, BUSCO gene alignments and trees, mitochondrial genome alignments and trees, GeMoMa annotations, snpden files, runs of homozygosity, RepeatMasker output, and PSMC output are publicly available on Figshare (https://doi.org/10.25573/data.c.5990545). This paper utilizes existing, publicly available data. These accession numbers for the datasets are listed in the key resources table. • All original code has been deposited at GitHub and is publicly available as of the date of publication. DOIs are listed in the key resources table. • Any additional information required to reanalyze the data reported in this paper is available from the lead contact upon request.
