# Supplementary material for: Whole genome analysis of clouded leopard species reveals an ancient divergence and distinct demographic histories
Source: iScience. 2022 Dec 9;25(12):105647. doi: 10.1016/j.isci.2022.105647 (PMC9801239; doi:10.1016/j.isci.2022.105647)
Supplement: Document S1. Figures S1–S3 and Tables S1–S3 [file mmc1.pdf]

iScience, Volume *25*

Supplemental information

Whole genome analysis of clouded leopard species reveals an ancient divergence and distinct demographic histories

Madeline G. Bursell, Rebecca B. Dikow, Henrique V. Figueiró, Olga Dudchenko, Joseph P. Flanagan, Erez Lieberman Aiden, Benoit Goossens, Senthilvel K.S.S. Nathan, Warren E. Johnson, Klaus-Peter Koepfli, and Paul B. Frandsen

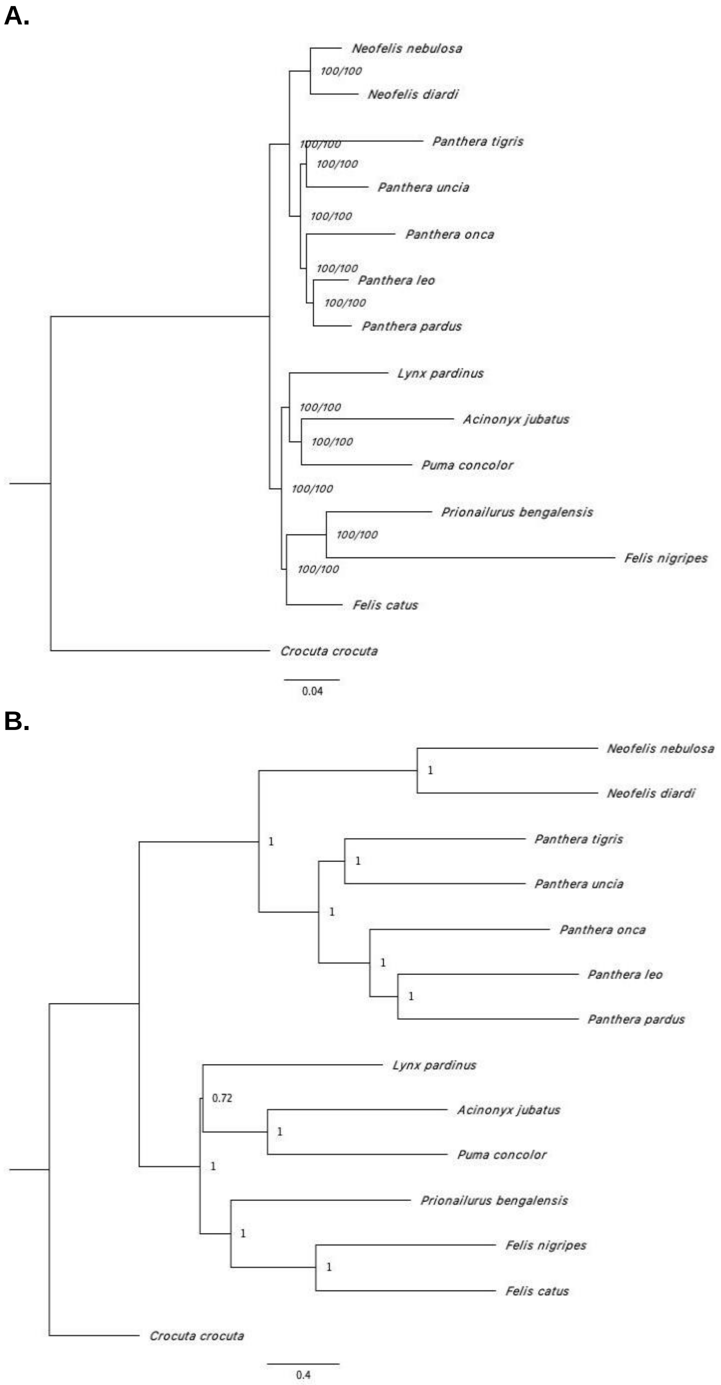

Figure S1: Maximum likelihood tree based on concatenated supermatrix and multispecies coalescent species tree generated using ASTRAL-III. Related to Figure 1 and STAR Methods dated phylogeny reconstruction. To create the time-tree phylogeny in Figure 1, we first estimated both a maximum likelihood tree based on a concatenated supermatrix[1–3] (A) and a multispecies coalescent tree estimated with ASTRAL_III v5.7.3[3,4] (B). The phylogenetic tree in Figure 1 was created using the multispecies coalescent tree as input. Values on each node on the maximum likelihood tree (A) are bootstrap and SH-aLRT support values while values on the multispecies coalescent tree (B) are local posterior probabilities.

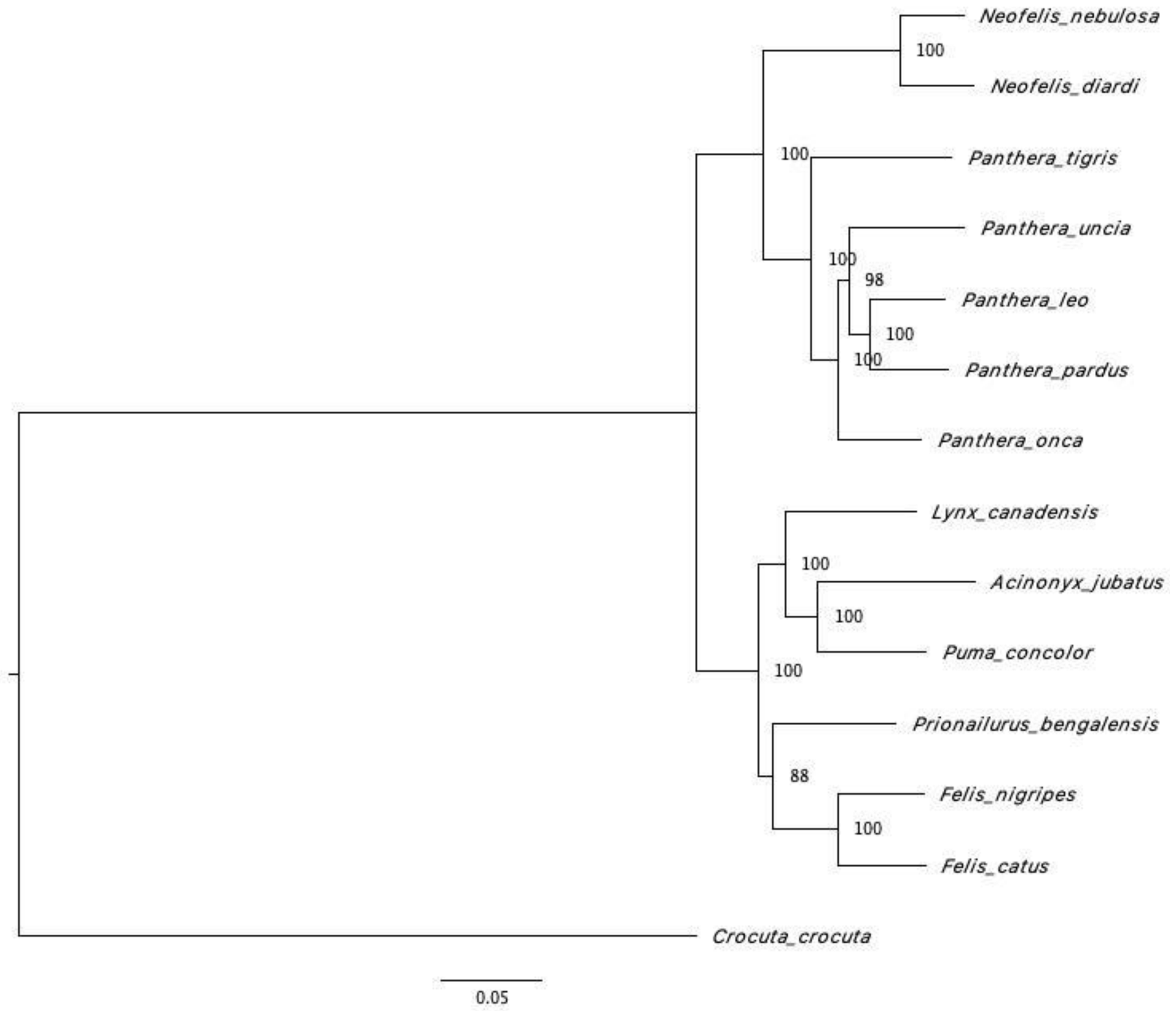

Figure S2: Mitochondrial genome phylogeny of 13 different species of felids and the spotted hyena (*Crocuta crocuta*) as an outgroup. Related to Figure 1. We estimated a maximum likelihood phylogenetic tree from mitochondrial genomes[1,3]. Phylogenetic bootstrap support values are shown for each node. (*Panthera tigris*: https://www.ncbi.nlm.nih.gov/nucleotide/NC_010642.1, *Panthera uncia*: https://www.ncbi.nlm.nih.gov/nucleotide/NC_010638.1, *Panthera leo*: https://www.ncbi.nlm.nih.gov/nucleotide/NC_028302.1, *Panthera pardus*: https://www.ncbi.nlm.nih.gov/nucleotide/NC_010641.1, *Panthera onca*: https://www.ncbi.nlm.nih.gov/nucleotide/NC_022842.1, *Lynx canadensis*: https://www.ncbi.nlm.nih.gov/nucleotide/NC_028313.1, *Acinonyx jubatus*: https://www.ncbi.nlm.nih.gov/nucleotide/NC_005212.1, *Puma concolor*: https://www.ncbi.nlm.nih.gov/nucleotide/NC_016470.1, *Prionailurus bengalensis*: https://www.ncbi.nlm.nih.gov/nucleotide/NC_028301.1, *Felis nigripes*: https://www.ncbi.nlm.nih.gov/nucleotide/NC_028309.1, *Felis catus*: https://www.ncbi.nlm.nih.gov/nucleotide/NC_001700.1)

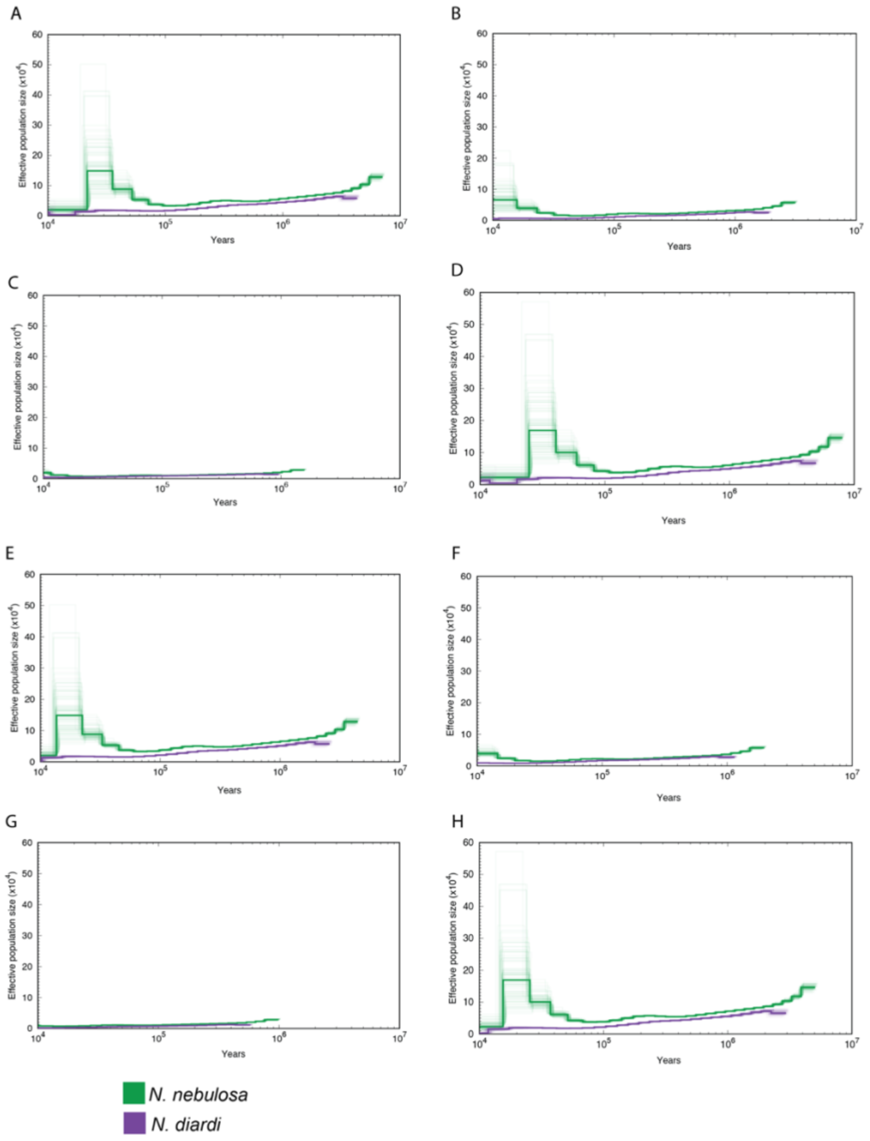

Figure S3: Plots of effective population size trajectories for *N. nebulosa* and *N. diardi* using different mutation rates and generation lengths for scaling. Despite variance among plots due to variable mutation rates, each plot shows increased effective population size of *N. nebulosa* compared with *N. diardi*. Related to Figure 2 and STAR Methods inference of historical effective population size with PSMC. **A)** Plot scaled with $2.22x10^{-9}$ mutation rate and generation length of 7.0 years for *N. nebulosa* and 7.3 years for *N. diardi*[5,6]. **B)** Plot scaled with $0.5x10^{-8}$ mutation rate and generation length of 7.0 years for *N. nebulosa* and 7.3 years for *N. diardi*[5,7]. **C)** Plot scaled with $1.0x10^{-8}$ mutation rate and generation length of 7.0 years for *N. nebulosa* and 7.3 years for *N. diardi*[5,8]. **D)** Plot scaled with $1.95x10^{-9}$ mutation rate and generation length of 7.0 years for *N. nebulosa* and 7.3 years for *N. diardi*[5,9]. **E)** Plot scaled with $2.22x10^{-9}$ mutation rate and generation length of 4.4 years for *N. nebulosa* and *N. diardi*[6,10]. **F)** Plot scaled with $0.5x10^{-8}$ mutation rate and generation length of 4.4 years for *N. nebulosa* and *N. diardi*[7,10]. **G)** Plot scaled with $1.0x10^{-8}$ mutation rate and generation length of 4.4 years for *N. nebulosa* and *N. diardi*[8,10]. **H)** Plot scaled with $1.95x10^{-9}$ mutation rate and generation length of 4.4 years for *N. nebulosa* and *N. diardi*[9,10].

	**Hi-C *Neofelis nebulosa***	**Scaffold *Neofelis nebulosa***	**Scaffold *Neofelis diardi***
Total # of base pairs	2,416,186,965	2,414,130,564	2,405,169,236
Total # of scaffolds	12,793	16,452	16,195
N10	221,013,172	4,357,083	5,170,218
N20	205,173,053	3,076,580	3,504,115
N30	167,137,124	2,291,304	2,607,972
N40	158,590,237	1,757,235	1,929,194
N50	147,111,411	1,375,641	1,390,302
L10	1	42	34
L20	2	108	93
L30	3	200	173
L40	4	321	281
L50	6	478	428
GC Content	41.65%	41.55%	41.32%
Median scaffold size	609	879	684
Mean scaffold size	188,868	146,738	148,513
Longest scaffold	239,539,317	9,244,571	11,465,388
Shortest scaffold	64	64	65

Table S1: Scaffold statistics for the HiC *Neofelis nebulosa* assembly and the scaffold assemblies for *Neofelis nebulosa* and *Neofelis diardi*. Related to STAR Methods whole genome assembly and annotation[11].

	**Hi-C *Neofelis nebulosa***	**Scaffold *Neofelis nebulosa***	**Scaffold *Neofelis diardi***
Total # of base pairs	2,408,254,133	2,408,254,138	2,387,726,162
Total # of contigs	84,944	84,480	122,201
N10	219,093	219,250	135,234
N20	158,361	158,551	99,112
N30	122,337	122,502	77,012
N40	96,841	97,014	61,207
N50	76,417	76,613	48,974
L10	829	828	1334
L20	2143	2141	3414
L30	3884	3880	6169
L40	6102	6094	9658
L50	8906	8891	14,026
GC Content	41.65%	41.65%	41.62%
Median contig size	10291	10421.5	8219
Mean contig size	28,351	28,506	19,539
Longest contig	651,517	651,517	512,408
Shortest contig	6	64	64

Table S2: Contig statistics for the Hi-C *Neofelis nebulosa* assembly and the scaffold assemblies for *Neofelis nebulosa* and *Neofelis diardi*. Related to STAR Methods whole genome assembly and annotation[11].

**Point estimate**	**Min**	**Max**	**Clade**	**Reference**
1.86	0.88	4.09	*Panthera pardus* + *P. leo*	Li et al. 2016
2.73	1.48	5.71	*P. onca* + (*P. pardus* + *P. leo*)	Li et al. 2016
2.67	11.14	5.92	*P. uncia* + *P. tigris*	Li et al. 2016
3.72	2.04	7.6	*Panthera*	Li et al. 2016
5.67	3.76	10.67	Pantherinae	Li et al. 2016
4.99	3.65	8.06	*Puma concolor* + *A. jubatus*	Li et al. 2016
8.76	6.19	12.52	Puma lineage + Lynx lineage	Li et al. 2016
4.23	2	7.16	*Felis*	Li et al. 2016
7.25	5.16	10.61	*Prionailurus* + *Felis*	Li et al. 2016
8.17	5.83	11.86	Felinae	Li et al. 2016
11.46	7.91	16.35	Felidae	Li et al. 2016

Table S3: Calibration priors used for divergence times estimates of *Neofelis*. Related to Figure 1C and STAR Methods dated phylogeny reconstruction. These calibration priors were used as input to MCMCtree[12] to create the time-scaled phylogeny in Figure 1. All dates were derived from the results from Li et al. 2016[13].

Supplemental References

1. Katoh, K., Misawa, K., Kuma, K., and Miyata, T. (2002). MAFFT: a novel method for rapid multiple sequence alignment based on fast Fourier transform. Nucleic Acids Res. *30*, 3059–3066. 10.1093/nar/gkf436
2. Kück, P., and Meusemann, K. (2010). FASconCAT: Convenient handling of data matrices. Mol. Phylogenet. Evol. *56*, 1115–1118. 10.1016/j.ympev.2010.04.024
3. Nguyen, L.-T., Schmidt, H.A., Haeseler, A. von, and Minh, B.Q. (2015). IQ-TREE: A Fast and Effective Stochastic Algorithm for Estimating Maximum- Likelihood Phylogenies. Mol. Biol. Evol. *32*, 268–274. 10.1093/molbev/msu300
4. Zhang, C., Rabiee, M., Sayyari, E., and Mirarab, S. (2018). ASTRAL-III: polynomial time species tree reconstruction from partially resolved gene trees. BMC Bioinf. *19*, 153. 10.1186/s12859-018-2129-y
5. Pacifici, M., Santini, L., Di Marco, M., Baisero, D., Francucci, L., Grottolo Marasini, G., Visconti, P., and Rondinini, C. (2013). Generation length for mammals. Nat. Conserv. *5*, 89–94. 10.3897/natureconservation.5.5734
6. Kumar, S., and Subramanian, S. (2002). Mutation rates in mammalian genomes. Proc. Natl. Acad. Sci. *99*, 803–808. 10.1073/pnas.022629899
7. Cho, Y.S., Hu, L., Hou, H., Lee, H., Xu, J., Kwon, S., Oh, S., Kim, H.-M., Jho, S., Kim, S., et al. (2013). The tiger genome and comparative analysis with lion and snow leopard genomes. Nat. Commun. *4*, 2433. 10.1038/ncomms3433
8. Figueiró, H.V., Li, G., Trindade, F.J., Assis, J., Pais, F., Fernandes, G., Santos, S.H.D., Hughes, G.M., Komissarov, A., Antunes, A., et al. (2017). Genome-wide signatures of complex introgression and adaptive evolution in the big cats. Sci. Adv. *3*, e1700299. 10.1126/sciadv.1700299
9. Liu, G.E., Matukumalli, L.K., Sonstegard, T.S., Shade, L.L., and Van Tassell, C.P. (2006). Genomic divergences among cattle, dog and human estimated from large-scale alignments of genomic sequences. BMC Genomics *7*, 140. 10.1186/1471-2164-7-140
10. Fazio, J., Andrews, J. (2018). Clouded leopard (*Neofelis nebulosa*) AZA Species Survival Plan, Yellow Program
11. Trizna M. 2020. assembly_stats 0.1.4. Zenodo. Available from: 10.5281/ Zenodo.3968775
12. Yang, Z. (2007). PAML 4: Phylogenetic Analysis by Maximum Likelihood. Mol. Biol. Evol. *24*, 1586–1591. 10.1093/molbev/msm088
13. Li, G., Davis, B.W., Eizirik, E., Murphy, W.J. (2016). Phylogenomic evidence for ancient hybridization in the genomes of living cats (Felidae). Genome Res *26*, 1-11. 10.1101/gr.186668.114
